# Patient outcomes following positive depression screening in a postpartum clinic: A single center retrospective cohort study

**DOI:** 10.18103/mra.2026.0389

**Published:** 2026-07-31

**Authors:** Aleesha Jasmin Jethwa, Nan Guo, Natasha Harrison, Dipro Chakraborty, Kimberly Mendoza, Natsumi Kii, Brendan Carvalho, Danielle Mari Panelli, Pervez Sultan

**Affiliations:** 1 Department of Anesthesiology, Perioperative and Pain Medicine, Stanford University School of Medicine, USA; 2 Department of Anesthesiology, Sapporo Medical University, Japan; 3 Department of Obstetrics and Gynecology, Stanford University School of Medicine, USA

## Abstract

**Background:**

Depression screening using the Edinburgh Postnatal Depression Scale is commonly performed at the 6-week postpartum visit, yet subsequent referral patterns and clinical outcomes are incompletely characterized.

**Methods:**

A single center cohort study was conducted using electronic health records of patients with a completed depression screen at the 6-week postpartum clinic between 2014–2023. Exposure was categorized as screen negative (Edinburgh Postnatal Depression Scale 0–9) or screen positive (Edinburgh Postnatal Depression Scale ≥10) for depressive symptoms. Primary outcome: the proportion of new mental health referrals made (to social work and/or psychiatry services) following the 6-week postpartum depression screen up to 1 year after childbirth. Secondary outcomes: the proportion of patients referred to each specialty after 6 weeks and during the peripartum period (antenatal to 1 year postpartum); patient variables associated with specialty referral from 6 weeks to 1 year postpartum using a multivariable logistic regression model; timing of postpartum referrals after 6-week depression screening; and psychiatric (medications, hospitalizations, mental health diagnoses) and infant associated outcomes within 1 year of childbirth.

**Results:**

Of 41,790 deliveries, 8,155 patients met inclusion criteria. 12.6% received a new mental health referral following 6-week postpartum depression screening up to 1 year after childbirth. After screening, social work and psychiatry referrals occurred in 4.7% and 3.7% of negative-screened patients and 37.1% and 19.5% of positive-screened patients, respectively. Across the peripartum period, social work and psychiatry referrals occurred in 8.6% and 17.5% of negative-screened patients, and 42.0% and 42.5% of positive-screened patients, respectively. Variables associated with referrals (6 weeks to 1 year postpartum) included divorced, separated and widowed marital status, ASA grade ≥3, delivery of a non-viable fetus, neonatal intensive care hospitalization, psychiatric diagnosis, and medication use. Referrals within 2 months postpartum were more frequent following positive screening compared to negative screening; Social Work (84% vs 49%); Psychiatry (53% vs 32%). Psychiatric medications, hospitalizations, mental health diagnoses and infant associated outcomes were more frequent following positive screening.

**Conclusion:**

Positive postpartum depression screening was associated with increased referrals to social work and psychiatry services; however, a critical gap persists between positive screening and specialty referral, diagnosis, and initiation of treatment. Findings support longitudinal mental health screening across the peripartum period.

## Introduction

Postpartum depression (PPD) occurs in approximately 15% of individuals following childbirth in the US, and is recognized as a major public health issue associated with increased maternal and neonatal morbidity and mortality.^1–5^ PPD impairs maternal infant bonding, is associated with maternal suicide and also has negative effects on infant development, such as lower intelligence quotient, delayed language development, and behavioral problems.^6^

Given the individual, societal and economic burden associated with PPD, professional guidance from the American College of Obstetricians and Gynecologists (ACOG) endorses routine screening with a validated tool, such as the Edinburgh Postnatal Depression Scale (EPDS).^7–12^ Screening, when coupled with follow-up care, reduces both depressive symptoms and overall prevalence of PPD.^13^ However maternal and neonatal outcomes, particularly regarding specialty referrals, commencement of pharmacotherapy and subsequent neonatal diagnoses following positive PPD screening are under explored.^13,14^ Internationally, a 26,000 patient audit from Australia linked positive antenatal EPDS to adverse obstetric and neonatal events, but provided no information on mental-health referrals, medication use, or subsequent associated International Classification of Diseases, Tenth Revision (ICD-10) diagnoses.^15^ The largest US single-site study of 10,000 postpartum women revealed higher rates of bipolar-spectrum illness and suicidal ideation among screen positive patients but did not report downstream referrals, treatment or follow-up care.^16^ Addressing this knowledge gap would be invaluable for staff and patient education and could help improve screening program compliance and implementation.^13,17^ Understanding clinical outcomes following positive PPD screening could also help primary care service planning, resource and staffing allocation, and help generate actionable targets for quality-improvement initiatives and guideline refinement.^13^

The primary aim of this study was to determine the proportion of new mental health referrals made (to social work and/or psychiatry services) from the 6-week postpartum EPDS screen up to 1 year after childbirth. Secondary aims were to determine the proportion of patients referred to each specialty after 6 weeks and during the peripartum period (antenatal to 1 year postpartum); patient variables associated with specialty referral from 6 weeks to 1 year postpartum using a multivariable logistic regression model; timing of postpartum referrals after 6-week EPDS screening; and psychiatric (medications, hospitalizations, ICD-10 mental-health diagnosis codes) and infant associated outcomes within 1 year of childbirth.

## Methods

Using STrengthening the Reporting of OBservational studies in Epidemiology (STROBE) guidelines, a retrospective cohort study was conducted following Institutional Review Board (IRB) approval.^18^ People delivering at a US academic healthcare system between 2014 and 2023 who completed EPDS screening during the postpartum clinic appointment (typically done 4–8 weeks postpartum, hereafter “6 weeks postpartum”), were included in the study. The annual rate of delivery at this center is 5,200 and EPDS screening at the postpartum clinic has been conducted since 2014.

Inclusion criteria for participants included the following: age ≥ 18 years at the time of childbirth; delivery of a live or non-viable fetuses after gestational age ≥ 20 weeks, via any delivery mode at the hospital system; completion of all items of the postpartum EPDS screening tool (completed between 4 weeks 0 days and 8 weeks 0 days postpartum) and postpartum care delivered within the healthcare system. Exclusion criteria for participants were: no or incomplete documentation of the postpartum EPDS in the electronic health record (EHR); EPDS completed outside the specified time window (<4 weeks 0 days or >8 weeks 0 days postpartum); and EPDS score ≥10 while inpatient immediately following childbirth (which would typically prompt more urgent referrals occurring prior to our study period).

Data was collected from the EHR via an institutional clinical data warehouse containing live Epic (Verona, WI) data from an academic health system. The database model captures patient-specific variables including demographics, diagnoses, procedures, medication and laboratory test results, as well as structured content extracted from clinical notes. The data warehouse is hosted under a Business Associate Agreement (BAA). This platform aims to facilitate advanced data science research on sensitive clinical datasets by providing a secure and collaborative big data analytics environment.

Collected data included demographics (age, parity, race/ethnicity, insurance type, language), obstetric and childbirth variables (induction and/or augmentation of labor, duration of 1^st^ and 2^nd^ stage labor, mode of childbirth), psychiatric history (prior diagnoses, medications, hospitalizations, substance use), complications (including perineal trauma, postpartum hemorrhage, chorioamnionitis, admission to intensive care), use and type of labor analgesia, private, academic or community obstetric services, and neonatal outcomes (birth weight, hospitalization in a neonatal intensive care unit (NICU)). The ICD-10 codes used to populate psychiatric variables such as F53 (mental and behavioral disorders associated with the puerperium) are provided in Table 1.

The primary outcome of this study was the proportion of new mental health referrals made (to social work and/or psychiatry services) from the 6-week postpartum EPDS screen up to 1 year after childbirth. Secondary outcomes were the proportions referred to each specialty after 6 weeks and during the peripartum period (antenatal to 1 year postpartum); patient variables associated with specialty referral from 6 weeks to 1 year postpartum using a multivariable logistic regression model; timing of postpartum referrals after 6-week EPDS screening; and psychiatric (medications, hospitalizations, ICD-10 mental-health diagnosis codes) and infant associated outcomes within 1 year of childbirth.

### Statistical methods

Continuous variables were compared using ANOVA for normally distributed variables, and Mann Whitney U test for non-parametric comparisons. An uncorrected chi-square test was used to compare the frequencies of categorical variables between the two groups (EPDS score <10 vs ≥10) or Fisher’s exact test. Data are presented as mean (standard deviation, SD), median [interquartile range, IQR] for continuous variables, or number (percentage) for categorical variables. Univariable and multivariable log-binomial models were used to assess the risk factors for referral to social work or psychiatry specialties. Multivariable models were adjusted for potential confounders with a p-value < 0.2 considered significant in univariable analysis. Missing data was assessed for all analytic variables.

Data was assumed to be missing at random (MAR) conditional on observed covariates. Missing data were handled using multiple imputation by chained equations (MICE) under MAR assumption. Five imputed datasets were generated in STATA 19 using predictive mean matching for continuous and multinomial logistic regression for categorical variables. Estimates were combined using Rubin’s rules. A sensitivity analysis was done using complete case analysis. All analyses were performed using two-tailed hypothesis testing with a P-value of less than 0.05 as statistically significant. All statistical analyses were performed using STATA (Version 19.5, College Station, TX).

## Results

**Figure 1:**
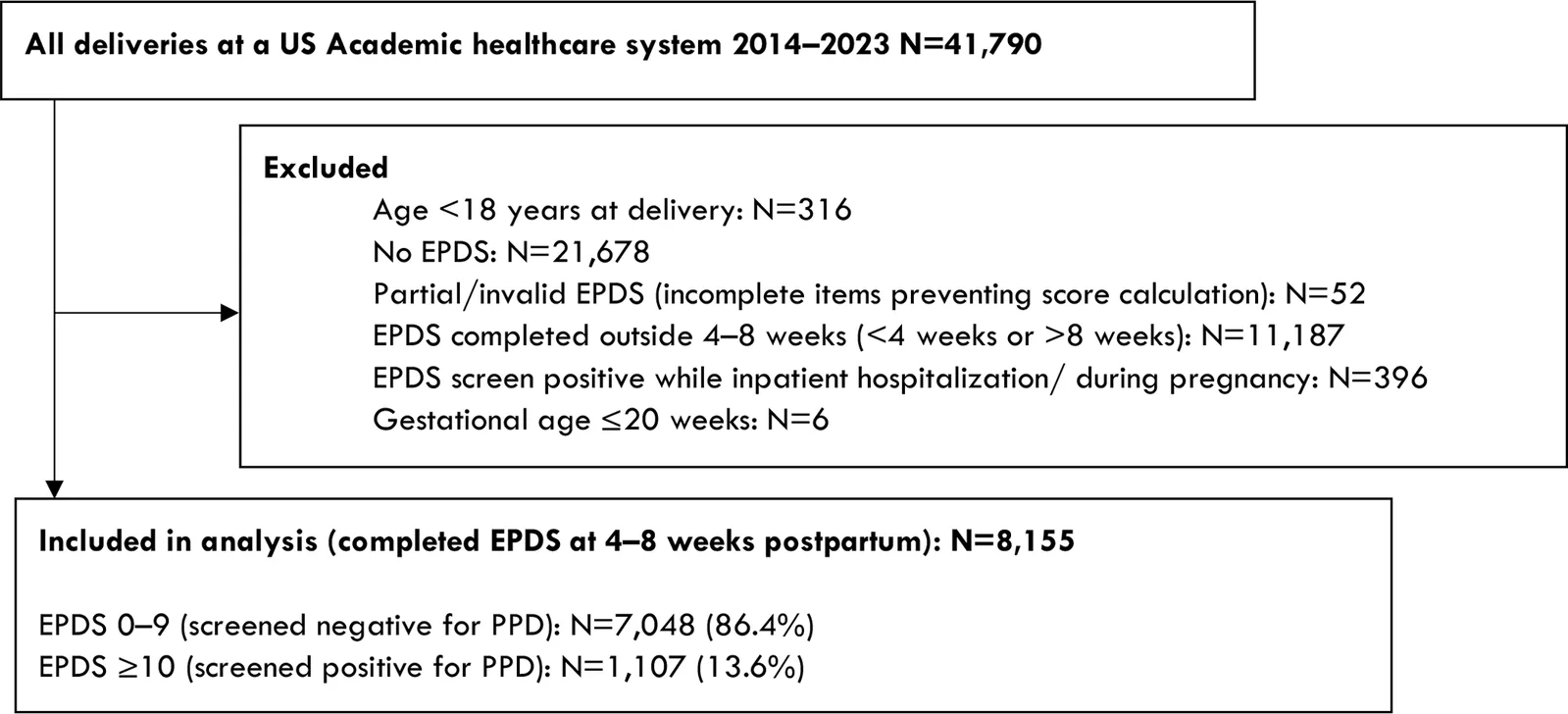
STROBE Diagram Flow of participants for the 6-week postpartum EPDS analysis and psychiatric outcomes subset at a quaternary healthcare system 2014–2023. Of 41,790 deliveries, 8,155 had a fully completed EPDS at 4–8 weeks postpartum and were included in the primary analysis; 1,107 (13.6%) screened positive (EPDS ≥10). EPDS = Edinburgh Postnatal Depression Scale; PPD = postpartum depression. Exclusions were applied sequentially. A positive screen was defined as EPDS ≥10. The proportion of missingness ranged from 0.2% to 13.4% (Table 2).

41,790 patients delivered at a quaternary healthcare system between 2014 and 2023. A 6-week postpartum EPDS score was collected from 8,155 patients and included in the analysis. The STROBE flow diagram is shown in Figure 1.

Table 3 summarizes the demographic, obstetric, anesthesia, psychiatry, and neonatal variables in patients that screened negative and positive for PPD.

Overall, 1,107 (13.6%) screened positive for PPD (EPDS score ≥10) from 6 weeks to 1 year postpartum. Median (IQR) EPDS score in the patients that screened negative and positive were 3 [1–5], and 12 [11–16], respectively. Patients with an EPDS score ≥10 were more frequently non-White, Hispanic, single, ASA grade ≤3, multiparous, delivered a non-viable fetus, delivered a baby with very low birth weight, and had a psychiatric diagnosis following postpartum EPDS screening.

Table 4 shows the proportion of new mental health referrals made to social work and/or psychiatry services from the 6-week postpartum EPDS screen (positive or negative) up to 1 year after childbirth (12.6%; 1024/8155). 45% and 7.5% of patients with a positive and negative EPDS screen were referred, respectively.

Table 5 shows the overall number of referrals to social work and/or psychiatry services during the peripartum period (antenatally up to one year following childbirth) and an associated EPDS screen conducted postpartum. Patients were more likely to be referred to either social work or psychiatry services antenatally regardless of EPDS screening status. 59% and 21% of patients with a positive and negative EPDS screen were referred to either of these services, respectively.

Table 6 shows the multivariable regression multiple imputed model used to identify factors which are independently associated with referral to social work or psychiatric services. Risk factors for referral to these services included positive EPDS screening score ≥10, widowed, divorced, and separated marital status, ASA grade ≥3, delivery of a non-viable fetus, hospitalization of baby in NICU, a psychiatric diagnosis and psychiatric medication use.

A complete case analysis was also performed (Table 7) with consistent findings to the multiple imputed model, except for suicide attempt being an additional risk factor.

Table 8 shows the distribution and timings of patient referrals made to social work and psychiatry services over a 1-year period following childbirth. For patients who screened positive (EPDS score ≥10) and were referred, most referrals (social work 84.4%; 347/411 and psychiatry services 53.2%; 115/216) occurred within the first two months postpartum. A minority of referrals occurred after 6 months postpartum (social work 2.7%; 11/411, psychiatry services 9.3%; 20/216). For patients who screened negative (EPDS 0–9) and were referred, most referrals also occurred within the first 2 months postpartum (social work 48.9%; 161/329 and psychiatry services 32.1%; 84/262). However, more than 20% of referrals occurred after 6 months postpartum (social work 21.8%; 72/329, psychiatry services 31.7%; 83/262).

Table 9 shows the outcomes following a postpartum EPDS positive and negative screening. More patients with an EPDS score ≥10 were referred to a social worker or medical psychiatry services postpartum, diagnosed with a psychiatric condition, started on psychiatric medication, hospitalized by psychiatry, and/or had a child requiring admission to hospital or a new disease diagnosis.

## Discussion

In our retrospective cohort study, we identified 12.6% of patients with a documented referral to social work and/or psychiatry services following the 6-week postpartum EPDS screen up to 1 year after childbirth. Of the 13.6% of patients who screened positive for PPD (EPDS ≥10), we noted a critical gap; only 45% were referred to social work and/or psychiatry services. While universal screening enables the identification of those at risk of PPD, our findings show that there is a discrepancy between detection and initiation of care, and a potential missed opportunity for improving maternal morbidity and outcomes.^13,14^

The referral patterns across the peripartum period show that although there were more referrals in the negative EPDS group overall, the timing of referral may be more informative than the absolute number of referrals. Most patients with a negative EPDS screen at 6 weeks who were seen by psychiatry or social work services had been referred antenatally rather than postpartum (951 vs 526). This may suggest that antenatal assessment identified some at-risk patients before postpartum screening, and highlights the importance of antenatal monitoring and referral pathways.^12,17^

Patient variables associated with specialty referral from 6 weeks to 1 year postpartum included widowed, divorced and separated marital status, ASA grade ≥3, delivery of a non-viable fetus, hospitalization of a baby in NICU, psychiatric diagnosis, and medication use. As referral data was collected solely from within the EHR, we do not know how involved patients were in the referral process. However, we suspect barriers to treating this population exist, not just at the individual level, but at interpersonal, institutional, community and policy levels, which may account for the discrepancy between the proportion of patients that screened positive and the proportion that were seen by specialty services.^14,20,21^ The majority of women who experience PPD do not enroll in psychiatry services following referral.^22^ Individual barriers such as lack of knowledge, time management concerns, beliefs on seeking help and resistance to medications play a role, however institutional level barriers such as inadequate referral protocols or Medicaid coverage, community level barriers such as lack of childcare and transportation, and policy level barriers such as insurance status and poverty must also be considered.^14,20,21,23,24^

While the majority of referrals to social work and psychiatric services occurred within 2 months of childbirth for all screened patients at the postpartum clinic, our study demonstrated that over 20% of patients with a negative EPDS screen were referred to additional services following 6 months postpartum. This finding suggests that patients with a negative screen at 6 weeks may have ongoing social work and/or psychiatric needs in the first year postpartum that require exploration.

However, these later referrals may not reflect a missed PPD diagnosis, but rather new or evolving symptoms. Prior data suggests that PPD symptoms may persist or emerge later in the 9–10 month postpartum period and are frequently unreported.^25^ Delayed referrals may therefore reflect motherhood transitions in the first year postpartum, different clinical phenotypes of PPD, potential false negative screening, under reporting of symptoms to healthcare providers due to stigma, or additional screening opportunities in pediatric and primary care settings at 1,2,4 and 6 months.^26–30^

We noted a difference in timing between social work and psychiatry referrals. For positive EPDS screened patients, social work referrals were initiated within the first two months postpartum (84%); however, medical psychiatry referrals were less frequently made within this period, with 53% occurring in the same period. The reasons for this may be intentional, as within current collaborative care models, social workers often serve as the first responder to maternal distress; however, it may also be due to systemic waitlist barriers.^14,20,31–34^

For patients who had a positive EPDS score at the postpartum clinic, approximately a third had a psychiatric diagnosis recorded, yet fewer than 10% of those with a confirmed psychiatric diagnosis were established on pharmacotherapy. This treatment gap is concerning given that the perinatal period carries a high risk of relapse and recurrence for women with mood disorders, particularly when maintenance pharmacotherapy is not established or is discontinued.^35–37^ While the EPDS is the current best measure to assess PPD, it is only a screening tool and further psychiatric evaluation is required to confirm PPD and determine if treatment is required.^10–13^ The reasons for this gap are likely multifactorial. Some factors may be healthcare dependent, such as local variation in referral pathways and access to psychiatry services, individual clinician experience and confidence initiating treatment, and whether the patient is within the health care system. ^14,20–22,38–40^ Other factors may relate to patient preferences and beliefs, including stigma, concerns about medication use while breastfeeding, and preferences for non-pharmacological treatment.^38,41–44^

We also noted a slightly increased proportion of infants within this screening category admitted to hospital or being diagnosed with a new condition within the year following childbirth. We know that untreated or undertreated PPD can lead to impaired maternal-infant bonding and infant consequences.^8,45^

While screening for EPDS is essential, it is insufficient unless there are robust pathways to connect patients to care.^13^ ACOG recommends integrating mental health care into obstetric practice, and a practical solution may be to create standardized and automated pathways instead of relying on passive referral systems that risk missing patients, such as collaborative care models, providing brief interventions for mothers, integrated into the obstetric clinic and therefore preventing patients from being lost to follow-up.^31,32,46,47^ Future work is needed to understand how best to support this population.

Key strengths of our study are the large sample size collected over a decade, therefore allowing us to collect a granular data set, which encompasses a diverse range of participant characteristics, as well as, being able to show both maternal and infant outcomes. We acknowledge that our findings are based on a single center which has individual screening practices and therefore may not be generalizable to the rest of the US.^48^ However our academic setting likely reflects comparatively good screening and referral practices compared to less well-resourced settings. Due to limitations of the EHR system and ICD-10 coding, misclassification is possible and we were unable to discern if patients attended their appointments and were adherent to treatment.^49^

## Conclusion

In summary, 12.6% of patients had a documented referral to social work and/or psychiatry services following the 6-week postpartum EPDS screen up to 1 year after childbirth. Positive EPDS screening was associated with increased social work and psychiatry referrals, psychiatric diagnosis, pharmacotherapy, hospitalization, and infant-associated outcomes. However, a critical gap persists between the proportion screening positive for depressive symptoms and those who receive specialty referral, diagnosis, and treatment. Peripartum referral patterns suggest that antenatal pathways may identify some at-risk patients before postpartum screening, while later referrals among patients who screened negative at 6 weeks suggest that social and psychiatric needs may arise later in the postpartum year. These findings support longitudinal mental health screening and follow-up across the peripartum period.

## Figures and Tables

**Table 1:** ICD-10 codes used for psychiatric variables

Variable	ICD-10 Code
Psychiatry diagnosis	F19: Mental and behavioral disorders due to psychoactive substance use F20-F29: Schizophrenia, schizotypal and delusional disorders F30-F39: Mood (affective) disorders F40-F48: Neurotic, stress-related and somatoform disorders F50-F59: Behavioral syndromes associated with physiological disturbances and physical factors F60-F69: Disorders of adult personality and behavior F80-F89: Disorders of psychological development F90-F98: Behavioral and emotional disorders with onset usually occurring in childhood and adolescence F99-F99: Unspecified mental disorder
Puerperium only	F53: Mental and behavioral disorders associated with the puerperium, not elsewhere classified
Domestic violence	Z91.41: Personal history of adult intimate partner abuse
Suicide attempt	T14.9: Suicide Attempt Z91.51: History of suicide attempt
Intentional self-harm	x60 - x83: Intentional Self-Harm Z91.5: Personal history of self-harm T36 - T50: Poisoning: Poisoning by adverse effect T51 - T65: Poisoning: Toxic effects of substances
Substance use	F10: Alcohol related disorders F11: Opioid related disorders F12: Cannabis related disorders F13: Sedative, hypnotic, or anxiolytic related disorders F14: Cocaine related disorders F15: Other stimulant related disorders F16: Hallucinogen related disorders F17: Nicotine dependence F18: Inhalant related disorders F19: Other psychoactive substance related disorders
Family history of mental and behavioral disorders	Z81: Family history of mental and behavioral disorders

ICD-10 = International Classification of Diseases, Tenth Revision. Listed codes were used to identify the corresponding psychiatric variables from electronic health record data.

ICD-10 codes for severe maternal morbidity data were chosen based on Centers for Disease Control and Prevention (CDC) recommendations.^19^

**Table 2:** Assessment of missing data

Variable	Missingness n (%)
Race	599 (7.0%)
Ethnicity	556 (6.5%)
Gestational age	18 (0.2%)
BMI at childbirth	119 (1.4%)
NICU	14 (0.2%)
ASA	359 (4.2%)
Health insurance	392 (4.6%)
Provider	700 (8.2%)
Parity	759 (8.9%)
Very low birth weight	46 (0.5%)
QBL	1140 (13.4%)
Worst pain after childbirth	34 (0.4%)

Data are presented as n (%). Percentages are calculated from the total analytic cohort (N = 8,155). ASA = American Society of Anesthesiologists physical status classification; BMI = Body Mass Index; NICU = Neonatal Intensive Care Unit; QBL = Quantitative Blood Loss.

**Table 3:** Patient Characteristics

	Negative PPD Screen (EPDS score 0–9) (N=7,048)	Positive PPD Screen (EPDS score ≥10) (N=1,107)	p-value

**Demographics**			

Age (years)	33.9±4.6	33.5±4.7	0.008

Body mass index, (kg/m^2^)			<0.001
<18.5	4 (0.1%)	0	
18.5 – 24.9	1288 (18.3%)	190 (17.2%)	
25 – 29.9	3146 (44.6%)	419 (37.9%)	
30 – 39.9	2198 (31.2%)	410 (37.0%)	
≥40	315 (4.5%)	69 (6.2%)	
Missing	97 (1.4%)	19 (1.7%)	

Race			0.003
White	2443 (34.7%)	329 (29.7%)	
Black	130 (1.8%)	27 (2.4%)	
Asian	2411 (34.2%)	383 (34.6%)	
AIAN or Pacific Islander	74 (1.1%)	20 (1.8%)	
Other	1496 (21.2%)	270 (24.4%)	
Unknown	494 (7.0%)	78 (7.1%)	

Ethnicity			<0.001
Non-Hispanic	5549 (78.7%)	799 (72.2%)	
Hispanic	1048 (14.9%)	228 (20.6%)	
Unknown	451 (6.4%)	80 (7.2%)	

Provider Service			<0.001
Unknown	557 (7.9%)	106 (9.6%)	
Private	611 (8.7%)	54 (4.9%)	
County	168 (2.4%)	24 (2.2%)	
Faculty	5712 (81.2%)	923 (83.4%)	

Marital Status			<0.001
Single	608 (8.6%)	157 (14.2%)	
Married/partner	6365 (90.3%)	937 (84.6%)	
Divorced/separated/widowed/unknown	75 (1.1%)	13 (1.2%)	

Health insurance			0.008
Private funding	5764 (81.8%)	862 (77.9%)	
Public funding	966 (13.7%)	184 (16.6%)	
Unknown	318 (4.5%)	61 (5.5%)	

**Anesthesia, obstetric and neonatal variables**			

ASA grade			0.232
1 or 2	5920 (84.0%)	927 (83.7%)	
≥3	816 (11.6%)	141 (12.7%)	
Missing	312 (4.4%)	39 (3.5%)	

Labor Analgesia/Anesthesia			0.121
Epidural	2099 (29.8%)	335 (30.3%)	
CSE	3387 (48.1%)	555 (50.1%)	
Spinal	676 (9.6%)	80 (7.2%)	
DPE	205 (2.9%)	39 (3.5%)	
No anesthesia	79 (1.1%)	10 (0.9%)	
Unknown	602 (8.5%)	88 (8.0%)	

Gravida	2 [1–3]	2 [1–3]	0.883

Parity	1 [1–2]	1 [1–2]	0.007

Gestational age, week	38.3±2.1	37.8±3.1	<0.001

Twin/multiple gestation	214 (3.0%)	49 (4.4%)	0.015

Delivery of a non-viable fetus	13 (0.2%)	10 (0.9%)	<0.001

Induction of Labor	2657 (37.7%)	445 (40.2%)	0.111

Delivery mode			0.718
Vaginal	4601 (65.3%)	709 (64.1%)	
Cesarean	2436 (34.6%)	396 (35.8%)	
Missing	11 (0.2%)	2 (0.2%)	

Indications for Cesarean Delivery			0.101
Arrest 1st stage	174 (7.1%)	35 (8.8%)	
Arrest 2nd stage	180 (7.4%)	31 (7.8%)	
Breech	205 (8.4%)	29 (7.3%)	
Multiple gestation	25 (1.0%)	9 (2.3%)	
Non-reassuring fetal status	359 (14.7%)	51 (12.9%)	
Other	362 (14.9%)	60 (15.2%)	
Prior Cesarean Delivery	473 (19.4%)	60 (15.2%)	
Missing	658 (27.0%)	121 (30.6%)	

Any Perineal Trauma/Tear	3784 (53.7%)	560 (50.6%)	0.054

QBL (ml)	335 [200–640]	350 [200–700]	0.025

HCT change/delta (preop. to postop.)	−4 [−6.4– −0.9]	−3.9 [−6.4 – 0]	0.223

Chorioamnionitis	965 (13.7%)	195 (17.6%)	<0.001

Length of stay postpartum (day)	2 [2–3]	2 [2–4]	0.005

Maternal ICU admission	85 (1.2%)	23 (2.1%)	0.018

Severe Maternal Morbidity (SMM)	301 (4.3%)	51 (4.6%)	0.609
Non-Transfusion SMM	273 (3.9%)	48 (4.3%)	0.462

Neonatal birth weight (g)	3250 [2900–3570]	3190 [2805–3536]	<0.001

Hospitalization at NICU	1045 (14.8%)	233 (21.1%)	<0.001

**Psychiatric variables**			

Psychiatry diagnoses			
All*	1138 (16.2%)	359 (32.4%)	<0.001
Only F53**	18 (0.3%)	6 (0.5%)	0.102

Suicide Attempt	8 (0.1%)	6 (0.5%)	0.001

Intentional Self Harm	156 (2.2%)	31 (2.8%)	0.225

Substance use	111 (1.6%)	29 (2.6%)	0.013

Family history	125 (1.8%)	32 (2.9%)	0.012

Domestic violence history	67 (1.0%)	17 (1.5%)	0.073

Psychological trauma history	69 (1.0%)	17 (1.5%)	0.092

Medications	541 (7.7%)	134 (12.1%)	<0.001
Anti-depressants	88 (1.3%)	25 (2.3%)	0.008
Anti-Obsessionals***	71 (1.0%)	21 (1.9%)	0.009
Anti-Anxiety	151 (2.1%)	41 (3.7%)	0.001
Sleep Medications	357 (5.1%)	89 (8.0%)	<0.001
Mood Stabilizers	21 (0.3%)	6 (0.5%)	0.189
Anti-Psychotics	15 (0.2%)	0	0.247

EPDS = Edinburgh Postnatal Depression Scale; ASA= American Society of Anesthesiologists physical status classification; F53 = ICD-10 code for Mental and behavioral disorders associated with the puerperium, not elsewhere classified; AIAN = American Indian and Alaska Native; CSE = Combined Spinal-Epidural; DPE = Dural Puncture Epidural; HCT = Hematocrit; QBL= Quantitative Blood Loss; NICU = Neonatal Intensive Care Unit

* All = Psychiatry diagnoses as per Table 1

** F53 = ICD-10 code for Mental and behavioral disorders associated with the puerperium, not elsewhere classified

*** Anti-obsessionals= clomipramine, fluoxetine, sertraline, paroxetine, fluvoxamine, citalopram, escitalopram

**Table 4:** Proportion of referrals to social work and psychiatry following EPDS screening at postpartum clinic up to 1 year following childbirth

	Negative screen (EPDS score 0–9) N=7,048	Positive screen (EPDS score ≥10) N=1,107	p-value
Social work referral	329 (4.7%)	411 (37.1%)	<0.001
Medical psychiatry Referral/consultation/visits	262 (3.7%)	216 (19.5%)	<0.001
Both social work and psychiatry referral	65 (0.9%)	129 (11.7%)	<0.001
Either social work or psychiatry referral	526 (7.5%)	498 (45.0%)	<0.001

Data are n (%). Percentages are calculated within each screen group (column). p-values compare positive versus negative screens (EPDS ≥10 vs <10) using the chi-square test or Fisher’s exact test, as appropriate. EPDS = Edinburgh Postnatal Depression Scale.

**Table 5:** Proportion of referrals to social work and psychiatry services during the peripartum period and associated EPDS screen postpartum

	Negative screen (EPDS score 0–9) N=7,048	Positive screen (EPDS score ≥10) N=1,107	p-value
Social work referral	605 (8.58%)	465 (42.0%)	<0.001
Medical psychiatry referral/ consultation/visit	1235 (17.5%)	459 (42.5%)	<0.001
Both social work and psychiatry referral	363 (5.2%)	271 (24.5%)	<0.001
Either social work or psychiatry referral	1477 (21.0%)	653 (59.0%)	<0.001

Data are n (%). Referrals were measured at any time from the antenatal period up to 1 year following childbirth. Percentages are calculated within each screen group (column). p-values compare positive versus negative screens (EPDS ≥10 vs <10) using the chi-square test or Fisher’s exact test, as appropriate. EPDS = Edinburgh Postnatal Depression Scale.

**Table 6:** Multivariable regression multiple imputed model identifying factors independently associated with referral to social work or psychiatric services

	Unadjusted		Adjusted	

	RR (95% CI)	p	RR (95% CI)	p

EPDS		<0.001		**<0.001**
Negative screen (Score 0–9)	Ref		**Ref**	
Positive screen (Score ≥10)	6.03 (5.43, 6.69)		**4.79 (4.27, 5.637)**	

Age	0.97 (0.96, 0.99)	<0.001	0.99 (0.98, 1.00)	0.066

Body mass index, (kg/m^2^)				
18.5 – 24.9	Ref		Ref	
25 – 29.9	1.04 (0.87, 1.24)	0.684	1.02 (0.86, 1.21)	0.794
30 – 39.9	1.57 (1.31, 1.87)	<0.001	1.20 (1.01,1.43)	0.035
≥40	2.30 (1.81, 2.93)	<0.001	1.26 (0.97, 1.65)	0.082

Race				
White	Ref		Ref	
Black	1.78 (1.31, 2.42)	<0.001	1.17 (0.88, 1.57)	0.288
Asian	0.77 (0.67, 0.90)	0.001	0.90 (0.78, 1.04)	0.147
AIAN or Pacific Islander	1.40 (0.91, 2.17)	0.128	0.97 (0.66, 1.43)	0.885
Other	1.29 (1.11, 1.49)	0.001	1.04 (0.87, 1.24)	0.652

Ethnicity		<0.001		0.459
Non-Hispanic	Ref		Ref	
Hispanic	1.66 (1.45, 1.89)		1.07 (0.90, 1.26)	

Provider Service		<0.001		**0.009**
Faculty/county	Ref		**Ref**	
Private	0.41 (0.28, 0.61)		**0.61 (0.42, 0.88)**	

Marital Status				
Single	Ref		Ref	
Married/partner	0.56 (0.48, 0.66)	<0.001	0.94 (0.80, 1.10)	0.448
Divorced/separated/widowed/unknown	1.27 (0.87, 1.86)	0.210	**1.43 (1.00, 2.03)**	**0.048**

Health insurance		<0.001		0.977
Private funding	Ref		Ref	
Public funding	1.60 (1.40, 1.84)		1.00 (0.85, 1.16)	

ASA grade		<0.001		**<0.001**
1 or 2	Ref		**Ref**	
≥3	1.89 (1.65, 2.17)		**1.33 (1.14, 1.54)**	

Parity		0.441		0.784
1	Ref		Ref	
≥1	0.95 (0.85, 1.08)		0.98 (0.86, 1.12)	

Gestational age		<0.001		0.780
37–43 week	Ref		Ref	
<37 weeks	1.74 (1.52, 2.00)		0.98 (0.82, 1.16)	

Twin/multiple gestation	1.16 (0.86, 1.56)	0.342	-	

Delivery of a non-viable fetus	3.84 (2.50, 5.91)	<0.001	**1.82 (1.20, 2.78)**	**0.005**

Perineal Trauma/Tear	0.76 (0.68, 0.85)	<0.001	0.96 (0.85, 1.08)	0.524

QBL (L)	1.13 (1.07, 1.20)	<0.001	1.01 (0.92, 1.11)	0.844

Chorioamnionitis	1.30 (1.12, 1.51)	<0.001	1.15 (1.00, 1.33)	0.085

Maternal ICU admission	2.33 (1.72, 3.15)	<0.001	1.15 (0.82, 1.62)	0.426

Very low birth weight	2.53 (2.02, 3.15)	<0.001	1.21 (0.91, 1.61)	0.180

Hospitalization in NICU	1.84 (1.62, 2.09)	<0.001	**1.25 (1.08, 1.44)**	**0.002**

Psychiatry diagnosis*	3.07 (2.74, 3.43)	<0.001	**1.95 (1.72, 2.20)**	**<0.001**

Suicide Attempt	5.16 (3.47, 7.65)	<0.001	1.41 (0.81, 2.44)	0.224

Intentional Self Harm	2.05 (1.59, 2.64)	<0.001	1.27 (0.98, 1.66)	0.073

Substance use	2.09 (1.56, 2.78)	<0.001	0.88 (0.64, 1.21)	0.440

Family history	1.86 (1.39, 2.49)	<0.001	1.18 (0.89, 1.56)	0.256

Domestic violence history †	2.21 (1.55, 3.14)	<0.001	-	

Psychological trauma history †	2.25 (1.60, 3.18)	<0.001	-	

Medications **	2.52 (2.20, 2.89)	<0.001	**1.45 (1.26, 1.67)**	**<0.001**

Estimates are from univariable and multivariable log-binomial regression models using five multiply-imputed datasets (multiple imputation by chained equations), combined using Rubin’s rules. Variables with p < 0.2 in univariable analysis were entered into the multivariable model. The adjusted model was adjusted for all variables shown. Values are RR (95% CI). Bold indicates statistical significance (p < 0.05). RR = Relative Risk; CI = Confidence Interval; Ref = Reference Category; EPDS = EPDS = Edinburgh Postnatal Depression Scale; BMI = Body Mass Index; ASA = American Society of Anesthesiologists physical status classification; AIAN = American Indian and Alaska Native; QBL = Quantitative Blood Loss; ICU = Intensive Care Unit; NICU = Neonatal Intensive Care Unit.

† Removed from the adjusted model due to multicolinearity.

* As defined in Table 1

** Medications include antidepressants, anti-obsessionals, anti-anxiety agents, sleep medications, mood stabilizers, and antipsychotics

**Table 7:** Multivariable regression identifying factors independently associated with referral to social work or psychiatric services: complete case analysis

	Unadjusted		Adjusted	

	RR (95% CI)	p	RR (95% CI)	p

EPDS		<0.001		**<0.001**
Negative screen	Ref		**Ref**	
Positive screen	6.03 (5.43, 6.69)		**4.96 (4.29, 5.73)**	

Age	0.97 (0.96, 0.99)	<0.001	0.99 (0.98, 1.01)	0.423

Body mass index, (kg/m^2^)				
18.5 – 24.9	Ref		Ref	
25 – 29.9	1.04 (0.87, 1.25)	0.667	0.91 (0.74, 1.12)	0.376
30 – 39.9	1.57 (1.32, 1.88)	<0.001	1.11 (0.89, 1.37)	0.358
≥40	2.31 (1.82, 2.93)	<0.001	1.16 (0.82, 1.63)	0.397

Race				
White	Ref		Ref	
Black	1.82 (1.34, 2.46)	<0.001	1.17 (0.83, 1.64)	0.364
Asian	0.76 (0.66, 0.89)	<0.001	0.86 (0.72, 1.03)	0.093
AIAN or Pacific Islander	1.43 (0.92, 2.23)	0.110	0.94 (0.56, 1.56)	0.805
Other	1.28 (1.11, 1.48)	0.001	1.03 (0.84, 1.27)	0.751

Ethnicity		<0.001		0.898
Non-Hispanic	Ref		Ref	
Hispanic	1.68 (1.47, 1.92)		0.99 (0.79, 1.23)	

Provider Service		<0.001		**0.001**
Faculty/county	Ref		**Ref**	
Private	0.36 (0.25, 0.51)		**0.42 (0.26, 0.67)**	

Marital Status				
Single	Ref		Ref	
Married/partner	0.56 (0.48, 0.66)	<0.001	**0.82 (0.68, 0.99)**	**0.040**
Divorced/ separated/widowed/unknown	1.27 (0.87, 1.86)	0.210	1.29 (0.82, 2.03)	0.276

Health insurance		<0.001		0.147
Private funding	Ref		Ref	
Public funding	1.61 (1.40, 1.85)		1.15 (0.95, 1.38)	

ASA grade		<0.001		**0.005**
1 or 2	Ref		**Ref**	
≥3	1.89 (1.65, 2.17)		**1.32 (1.09, 1.61)**	

Parity		0.491		0.865
1	Ref		Ref	
>1	0.96 (0.85, 1.08)		1.01 (0.87, 1.18)	

Gestational age		<0.001		0.463
37–43 week	Ref		Ref	
<37 weeks	1.75 (1.52, 2.01)		0.99 (0.81, 1.22)	

Twin/multiple gestation	1.16 (0.86, 1.56)	0.342	-	

Delivery of a non-viable fetus	3.84 (2.50, 5.91)	<0.001	**2.07 (1.25, 3.42)**	**0.005**

Perineal Trauma/Tear	0.76 (0.68, 0.85)	<0.001	1.04 (0.88, 1.22)	0.648

QBL (L)	1.14 (1.07, 1.21)	<0.001	0.96 (0.82, 1.13)	0.620

Chorioamnionitis	1.30 (1.12, 1.51)	<0.001	1.07 (0.89, 1.30)	0.453

Maternal ICU admission	2.33 (1.72, 3.15)	<0.001	1.04 (0.63, 1.73)	0.868

Very low birth weight	2.54 (2.04, 3.17)	<0.001	1.28 (0.86, 1.92)	0.223

Hospitalization at NICU	1.84 (1.62, 2.09)	<0.001	**1.22 (1.01, 1.47)**	**0.039**

Psychiatry diagnoses	3.07 (2.74, 3.43)	<0.001	**1.96 (1.68, 2.28)**	**<0.001**

Suicide Attempt	5.16 (3.47, 7.65)	<0.001	**1.93 (1.08, 3.46)**	**0.026**

Intentional Self Harm	2.05 (1.59, 2.64)	<0.001	1.22 (0.88, 1.70)	0.231

Substance use	2.09 (1.56, 2.78)	<0.001	0.79 (0.52, 1.20)	0.275

Family history	1.86 (1.29, 2.49)	<0.001	1.10 (0.75, 1.61)	0.615

Domestic violence history †	2.21 (1.55, 3.14)	<0.001	-	

Psychological trauma history †	2.25 (1.60, 3.18)	<0.001	-	

*Medications	2.52 (2.20, 2.89)	<0.001	**1.38 (1.16, 1.65)**	**<0.001**

Estimates are from univariable and multivariable log-binomial regression models restricted to participants with complete data for all analytic variables (complete-case analysis), performed as a sensitivity analysis for the multiply-imputed primary model (Table 6). Variables with p < 0.2 in univariable analysis were entered into the multivariable model, which was adjusted for all variables shown. Values are RR (95% CI). Bold indicates statistical significance (p < 0.05). RR = Relative Risk; CI = Confidence Interval; Ref = Reference Category; EPDS = Edinburgh Postnatal Depression Scale; BMI = Body Mass Index; ASA = American Society of Anesthesiologists physical status classification; AIAN = American Indian and Alaska Native; QBL = Quantitative Blood Loss; ICU = Intensive care unit; NICU = Neonatal Intensive Care Unit. Psychiatric and related variables were identified using the ICD-10 codes listed in Table 1.

† Removed from the adjusted model due to multicolinearity.

* Medications include antidepressants, anti-obsessionals, anti-anxiety agents, sleep medications, mood stabilizers, and antipsychotics.

**Table 8:** Distribution and timing of patient referrals to additional care services (social work or psychiatry services) following EPDS screening at postpartum clinic appointment

	Social work referral N=740		Psychiatry referral/consultation/visit N=478	
Month following childbirth	Negative screen (EPDS 0–9) N=329	Positive screen (EPDS ≥10) N=411	Negative screen (EPDS 0–9) N=262	Positive screen (EPDS ≥10) N=216
<2 months	161 (48.9%)	347 (84.4%)	84 (32.1%)	115 (53.2%)
2–3 months	70 (21.3%)	47 (11.4%)	55 (21.0%)	67 (31.0%)
4–5 months	26 (7.9%)	6 (1.5%)	40 (15.3%)	14 (6.5%)
6–8 months	35 (10.6%)	7 (1.7%)	45 (17.2%)	16 (7.4%)
9–12 months	37 (11.2%)	4 (1.0%)	38 (14.5%)	4 (1.9%)

Data are n (%). Percentages are calculated within each column and represent the distribution of referral timing across the first year following childbirth. Time is measured from childbirth. EPDS = Edinburgh Postnatal Depression Scale; a positive screen was defined as EPDS ≥10.

**Table 9:** Outcomes following EPDS screening at postpartum clinic, up to 1 year following childbirth

	Negative screen (EPDS score 0–9) N=7,048	Positive screen (EPDS score ≥10) N= 1,107	p-value

Social work referral	329 (4.7%)	411 (37.1%)	<0.001

Medical psychiatry referral/consultation/visit	262 (3.7%)	216 (19.5%)	<0.001

Both social work and psychiatry referral	65 (0.9%)	129 (11.7%)	<0.001

Either social work or psychiatry referral	526 (7.5%)	498 (45.0%)	<0.001

Psychiatry diagnosis			
All*	479 (6.8%)	339 (30.6%)	<0.001
Only F53**	92 (1.3%)	201 (18.2%)	<0.001

Psychiatric medication	139 (2.0%)	33 (3.0%)	0.030

Child hospital admission after discharge or disease	744 (10.6%)	133 (12.0%)	0.021

Data are n (%). P-values compare positive versus negative screens (EPDS ≥10 vs <10) using the chi-square test or Fisher’s exact test, as appropriate. Referral and outcome events were measured from childbirth up to 1 year following childbirth. EPDS = Edinburgh Postnatal Depression Scale; ICD-10 = International Classification of Diseases, Tenth Revision.

* All psychiatry diagnoses, defined using the ICD-10 codes listed in Table 1.

** F53 = ICD-10 code for mental and behavioral disorders associated with the puerperium, not elsewhere classified; “Only F53” denotes patients whose sole psychiatric diagnosis was F53.
